# Reliability Analysis of the Electrical Control System of Subsea Blowout Preventers Using Markov Models

**DOI:** 10.1371/journal.pone.0113525

**Published:** 2014-11-19

**Authors:** Zengkai Liu, Yonghong Liu, Baoping Cai

**Affiliations:** College of Mechanical and Electrical Engineering, China University of Petroleum, Qingdao, Shandong, China; Universidad de Valladolid, Spain

## Abstract

Reliability analysis of the electrical control system of a subsea blowout preventer (BOP) stack is carried out based on Markov method. For the subsea BOP electrical control system used in the current work, the 3-2-1-0 and 3-2-0 input voting schemes are available. The effects of the voting schemes on system performance are evaluated based on Markov models. In addition, the effects of failure rates of the modules and repair time on system reliability indices are also investigated.

## Introduction

During offshore drilling, a subsea blowout preventer (BOP) is attached to the wellhead to seal an open wellbore, close the annular portion of the well around the drill pipe or casing, or cut through the drill pipe and then seal the well. A typical BOP system can enable certain well pressure tests as well as the injection and removal of fluid from the well through its lines [1]. Devastating consequences could be caused by failures of subsea BOP systems, for example, the Deepwater Horizon accident in the Gulf of Mexico on April 20, 2010. Eleven crew members died in the accident, and oil gushed out of the damaged well for months, which became the worst environmental disaster in US history [2]. Hence, ensuring the reliable and safe operation of subsea BOPs is essential. A distributed control system is employed to develop the electrical control system of a subsea BOP stack based on triple modular redundancy [3].

The issue with reliability of subsea BOP systems is of significance, which is not studied extensively enough. Failure modes and effects analysis method has been adopted for reliability analysis of a BOP [4]. The fault trees of subsea BOP system are built by collecting the data about subsea BOP failures and malfunctions [5]. However, the two methods are only suitable for non-repair systems and time element is not included [6]. For reliability analysis, Markov models are widely used in various fields. Duan et al. [7] propose an arithmetic coding based on Markov model as a general tool for joint encryption and compression. Ellis et al. [8] present a new Markov model for packet loss that can more accurately describe the characteristics of residential broadband links, and quantify the effectiveness of this model. Karami-Horestani et al. [9] propose a reliability model for a static VAr compensator based on Markov process. Jiang et al. [10] propose a Markov model and corresponding methods in term of human factors and other relating factors. Guo and Yang [11] present a new method to automatically develop Markov models for reliability assessment of safety instrumented systems, considering failure modes, self-diagnostic, restorations, common cause and voting. Lisnianski et al. [12] present a multi-state Markov model for a coal power generating unit and a technique for estimation of transition rates between the various generating capacity levels of the unit based on field observation. Tanrioven and Alam [13] present a method for reliability analysis of stand-alone PEM FCPPs using Markov model, which includes different states such as operation, derated, fully faulted or maintenance. Pil et al. [14] propose a Markov model to assess the reliability of reliquefaction systems for boil-off gas on LNG carriers with focus on redundancy optimization and maintenance strategies.

Owing to its flexibility for modeling, Markov method has been used to evaluate the redundant systems. A Markov-based model is presented for evaluating the performance of subsea BOP stack configurations and mounting types for control pods [15]. Wang et al. [16] perform the Markov analysis to analyze the redundant and non-redundant building cooling, heating, and power system, where the failure rate, the repair rate, the availability and the MTBF are deduced. Mendes et al. [17] develop a Markov model to analyze the reliability and determine the optimal interval between inspections of redundant system subjected to periodic inspections. Li [18] studies a k-out-of-n: G repairable system with n identical machines and R repairmen using Markov model, considering the redundant dependency and the multiple vacations policy for repairmen. Azaron ea al. [19] introduce a new method, by using Markov model and shortest path technique, for the reliability assessment of an l-dissimilar-unit non-repairable cold-standby redundant system, where each unit consists of many independent units arranged in any general configuration. Yu et al. [20] model the redundant dependency by embedding the dependence function into Markov models, describing a redundant system with dependent components.

The electrical control system of subsea BOP system is also a redundant system. In order to take into account the effects of different input voting schemes on system reliability indices, this paper develops Markov models for evaluation of the electrical control systems. A sensitivity analysis is performed to determine the influences of failure rates. The paper is structured as follows. Section 2 describes a subsea BOP electrical control system in detail. Section 3 presents the Markov models and analysis methods for the two input voting schemes. Section 4 delves into the effects of input voting schemes and variances of failure rates for each module. Section 5 summarizes the paper.

## System Description

**Figure 1:**
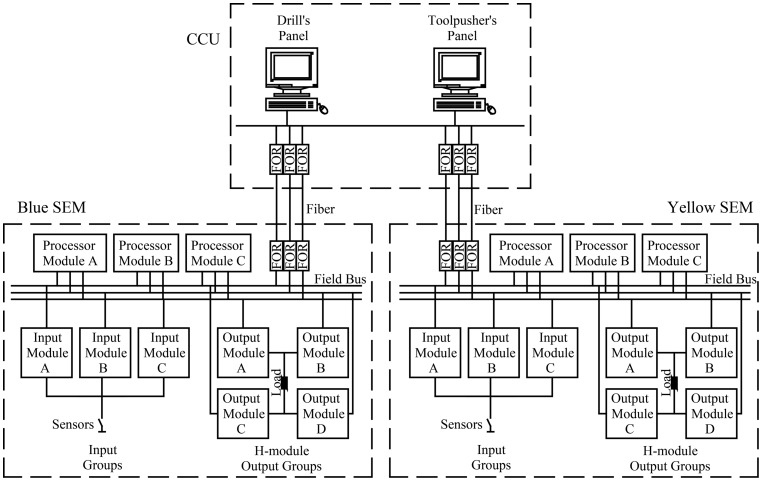
Architecture of subsea BOP electrical control system.

Fault tolerance technique is widely utilized in subsea BOP systems in order to improve reliability. The fault tolerant system has a redundancy, whereby minor faults can occur without halting normal operation [21]. For a typical subsea BOP stack, two annular BOPs and four ram BOPs are used [22]. A triple modular redundancy control system is developed to control a subsea BOP stack. The electrical control system mainly consists of a central control unit (CCU), a connecting umbilical cable, and subsea components, as shown in Figure 1. CCU is the central control point for controlling and monitoring system functions and communications. It has two main control panels, namely, Driller's panel and Toolpusher's panel. They can control all the functions of the subsea BOP system, which display push buttons designed to mimic the BOP stack and other functions. The Driller's panel is mounted by the driller on the rig floor for convenient access, whereas the Toolpusher's panel is located in a non-hazardous area away from the drill floor [23]. They are mainly industrial computers with equal functionality.

Two control pods are above the BOP stack, affording redundant control of subsea functions. Each pod contains a subsea electrical module (SEM), which is contained in domed containers made of thick steel under external water pressure [24]. One of the pods is functional, whereas the other one acts as a warm standby. Two complete independent subsea umbilical cables, including optical fibers and electrical wires, are used to transmit signals and electric power from the surface to the SEMs [25]. Fiber optic repeaters are used for transforming electrical and optical signals.

A redundancy technique is applied to develop the system in order to form a highly reliable electrical control system. The SEMs contain processor modules, input modules, and output modules, as well as other secondary components. Three processors are used to form a processor subsystem. Each processor runs the same application programs, processing data and sending command signals. The processor subsystem performs 2 out 3 voting, which means the system can hence detect and correct a single failure. The input and output modules are connected to three processors via three independent field buses. Output voting is performed in the H-module group, which is composed of two source output modules (A and B) and two sink modules (C and D). The source modules are connected in parallel on one side of the load, whereas the sink modules are connected in parallel on the other side. Module D is dependent on module A and module B and its logic is 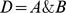. Therefore, modules A, B and C form a 2 out of 3 voting system. Data from the processors are voted in the H-module group, where one fault of the three modules can be masked.

Compared with output voting, input voting is accomplished by programs in the processors. Two input voting schemes adopted in this work, namely, the 3-2-1-0 scheme and the 3-2-0 scheme, are used to deal with failures in the modules. Aside from the input values, the configured Duplex and Default values may also be used to determine the final voted results. The Duplex value is used when only two input modules survive. It may be configured as any value according to the need in the field. The Default value will be provided directly to the application programs instead of a voted result; it may be configured as the last input value, or a specific value. For both schemes, when all three input values are available, the software performs two out of three voting runs, whereby the Duplex and Default values are not used. If any of the three input values are not available, the software will employ the configured Duplex value for performing two out of three voting runs, in order to replace the unavailable actual input. Configuration of the Duplex State value has to be in accordance with particular systems. The two input voting schemes have different ways to cope when two input modules fail. In the 3-2-0 input voting scheme, the Default value is applied to replace the remaining actual input value; however, the available input value will be the final result without voting in the 3-2-1-0 scheme. Thus, if two or more modules are not available, the electrical control system will fail in the 3-2-0 voting scheme. Unless all three input modules are unavailable, the system fails in the 3-2-1-0 voting scheme. Therefore, the main difference is that 3-2-0 voting scheme only can bear one fault and 3-2-1-0 scheme can bear two faults. Table 1 illustrates how the two voting schemes work, where the Duplex State and Default State are set as 0. ‘-’ means the input is unavailable and it will be replaced by the configured Duplex State value for voting.

**Table 1:** Voted results for the two schemes.

Input A	Input B	Input C	Voted results (3-2-1-0)	Voted results (3-2-0)
1	1	1	1	1
1	1	0	1	1
1	0	0	0	0
0	0	0	0	0
1	1	-	1	1
1	0	-	0	0
0	0	-	0	0
-	-	1	1	0
-	-	0	0	0
-	-	-	0	0

## System Modeling and Analysis

### System modeling

Although two different input voting schemes are available, the effects of different schemes on the reliability of the electrical control system are unknown. Therefore, a quantitative analysis about this issue is necessary. For the electrical control system of subsea BOPs, only control panels, processor modules, input modules, and output modules are covered in this paper; other secondary components of the system are considered completely reliable. If the surface components, namely, control panels fail, they will be repaired immediately without removing the BOP system from the water. The subsea components, namely input modules, output modules and processors will be pulled to the surface and repaired when the control system cannot work due to their failures.

**Figure 2:**
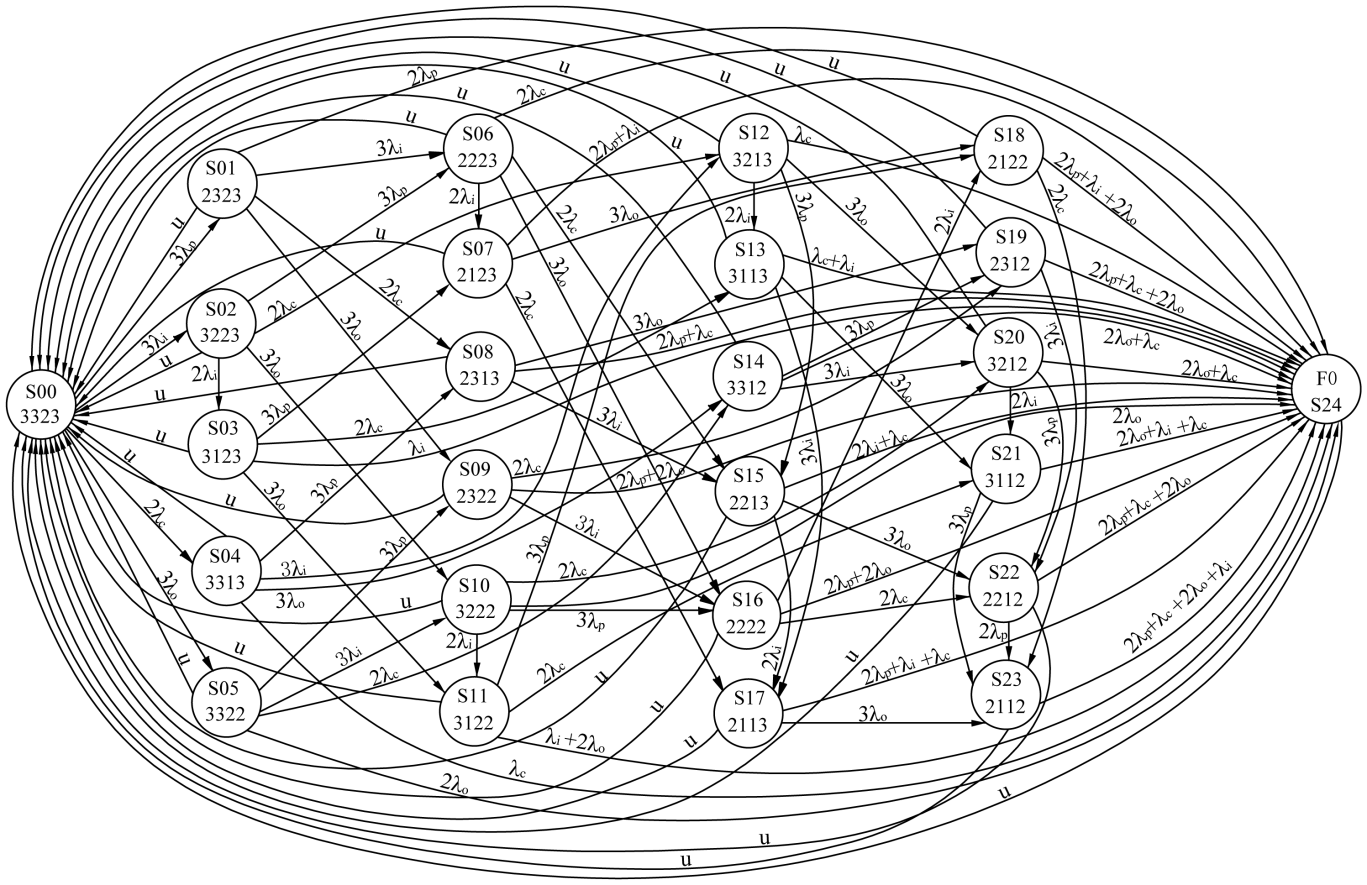
Markov model for 3-2-1-0 input voting scheme.

**Figure 3:**
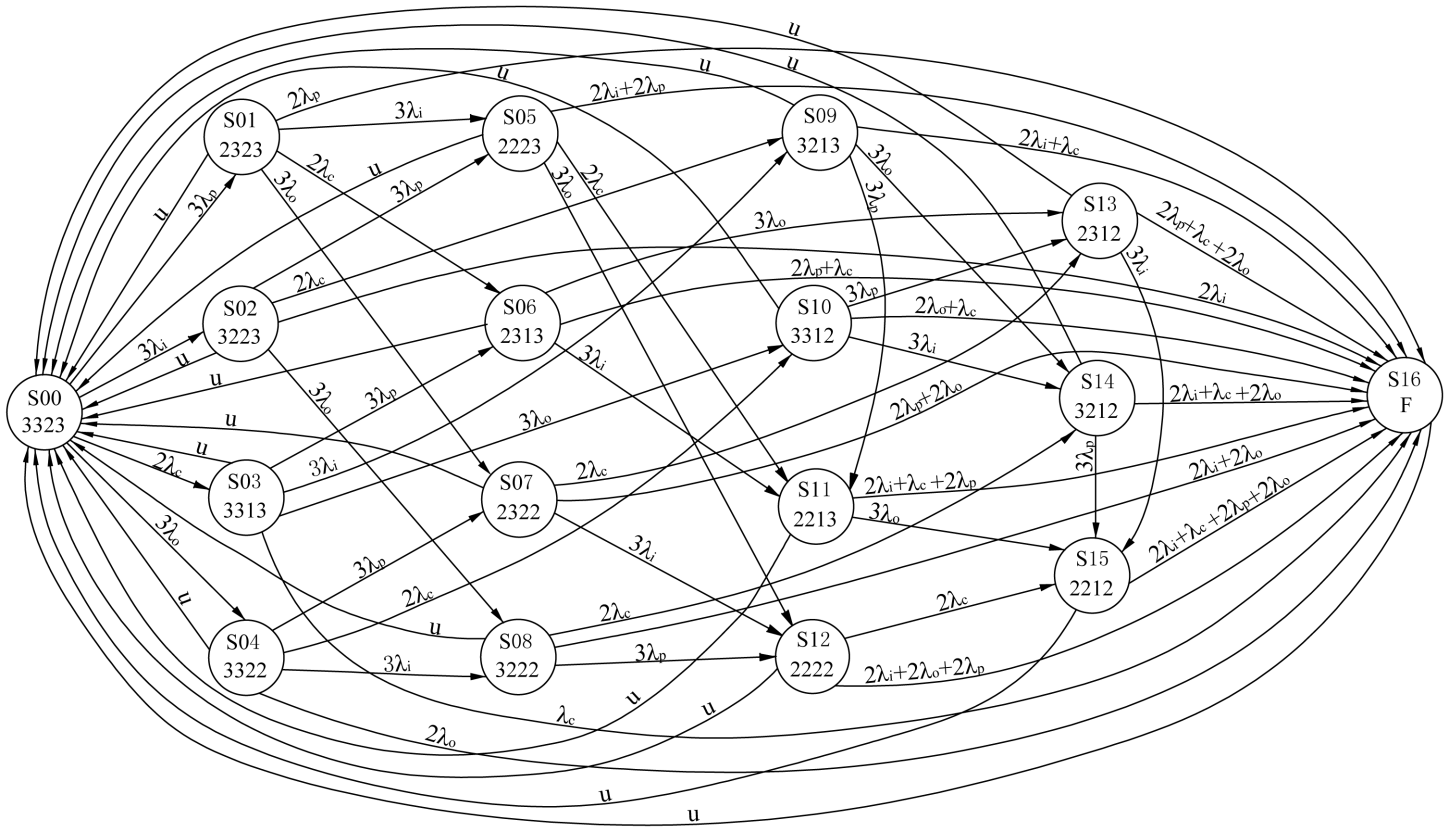
Markov model for 3-2-0 input voting scheme.

The state transition diagrams of the 3-2-1-0 and 3-2-0 input voting schemes are shown in Figure 2 and Figure 3, respectively. These schemes adhere to the input voting principle described in Section 2. In the figures, circles represent states, and directed arcs represent transitions caused by failures or repairs. A total of 25 states, denoted by S00-S24, are numbered in sequence in Figure. 2. The four digits below the state in the circle denote the number of the processors, input modules, control panels and output modules in normal operation, respectively. For example, the system starts with the perfect state, S00 and “3323”means 3 processors, 3 input modules, 2 control panels and 3 output modules work normally. In this state, when any failure occurs in the modules, the system moves to another state. The degrading states, S01 to S23, indicate continued performance of the system despite ongoing failures. In S02, for example, “3313” means only one control panel works, as the other one has failed and is being repaired, whereas the other modules are operating as expected. At this time, the system has tolerated a fault and is still viable. However, if another failure occurs in the working control panel, the control system will move from state S02 to state S24, which is the hazardous state. This state denotes that the control system is unavailable for users. According to the memoryless property of Markov model, the lifetime of components and repair time follow exponential distributions. The corresponding transition rate is marked on the directed arc form one state to another state. For example, 3λp is the transition rate from state S00 to S01. The definitions of transition rates and the values are listed in Table 2. For the sake of simplicity, repair rates of all the components are defined as *u*.

**Table 2:** Definition of transition rates.

Notation	Description	Values/h^-1^
*λp*	Failure rate of a processor module	1.820e-5
*λi*	Failure rate of an input module	9.798e-6
*λc*	Failure rate of a control panel	3.152e-5
*λo*	Failure rate of an input module	9.780e-6
*u*	Repair rate	1.388e-2

### Model analysis

In this section, the method to perform model analysis is proposed. The analysis process of the model for the 3-2-1-0 voting scheme is illustrated in detail as follows. The transition matrix of the model for 3-2-1-0 voting scheme are shown in Eq. (1), which is represented in simple forms.
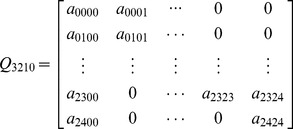
(1)


In the matrixes, element 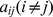 is the transition rate from state *i* to state *j*. Specially, the diagonal element 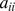 is obtained as follows:
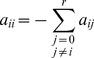
(2)


For example, 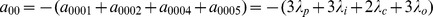.

#### Availability and steady-state availability

Availability is defined as the probability that the system is performing its required function at a given time. No operational time interval is involved. If the system is operational, it is available. History does not matter, that is, whether it has failed in the past and has been repaired or has been operating continuously without failure [26]. 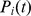 is the occupation probability of state *i* at time t. It can be calculate by Eq. (3). 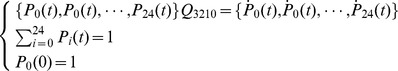
(3)where 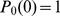 means that the system starts from state 0 at time 0. For the model of 3-2-1-0 voting scheme, only state S24 is unavailable for users. Therefore, the transient availability is given by



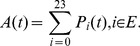
(4)The steady-state availability is defined as: 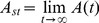
(5)


#### Reliability

Reliability describes the ability of a system or component to perform its required functions under stated conditions for a specified period of time [0, t]. No failures are allowed for an entire time interval. It is a function of failure probabilities and operating time interval. Therefore, to obtain the reliability of repairable systems, the transition arcs related to repairs after failures have to be omitted when to calculate the reliability. The transition matrix 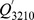 for reliability is obtained:
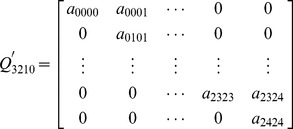
(6)



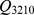
 is replaced by 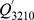 in Eq. (3) and reliability can be calculated by
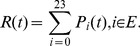
(7)


#### MTTF

Mean time to failure (MTTF) is an important reliability index for the system. It is defined as the mean time to the first system failure, when the system starts from the fully functioning state. The specific steps for calculating MTTF of 3-2-1-0 voting scheme are as follows [26]:

(1) 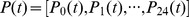 denotes the vector of state occupation probabilities at time t. The initial condition is 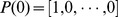 and it means that the system is in normal operation. The system will fail when it enters into state 24, which are defined as the absorbing states. Deleting the related elements of the absorbing states in transition matrix, the 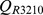 can be obtained:
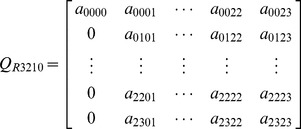
(8)


(2) 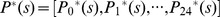 is defined as the Laplace transform of *P(t)*. Hence, Eq. (9) is derived.
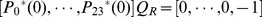
(9)


(3) Finally, the equation to compute MTTF is 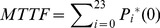
(10)


## Results and Discussion

### Availability

**Figure 4:**
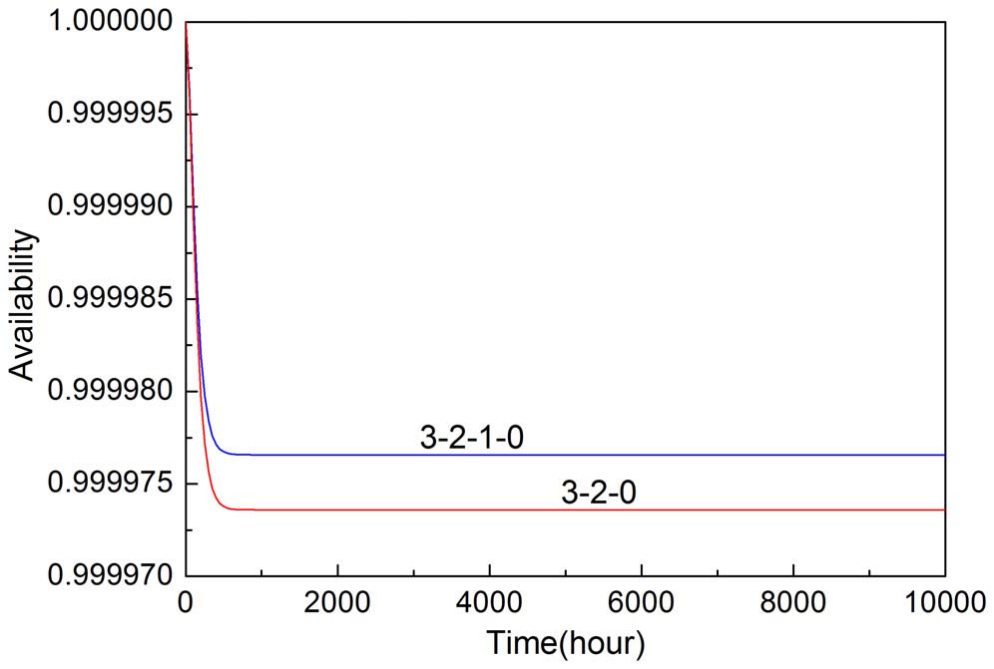
Availability of the systems in two input voting schemes.

By solving the Chapman-Kolmogorov Eq.(3), availability of the system with two input voting schemes can be obtained by Eq. (4), which is shown in Figure 4. It shows that availability decreases quickly in the first hundred hours and reaches steady-state values in about seven hundred hours. The 3-2-1-0 voting scheme has higher availability than 3-2-0 voting scheme.

### Steady-state availability

**Figure 5:**
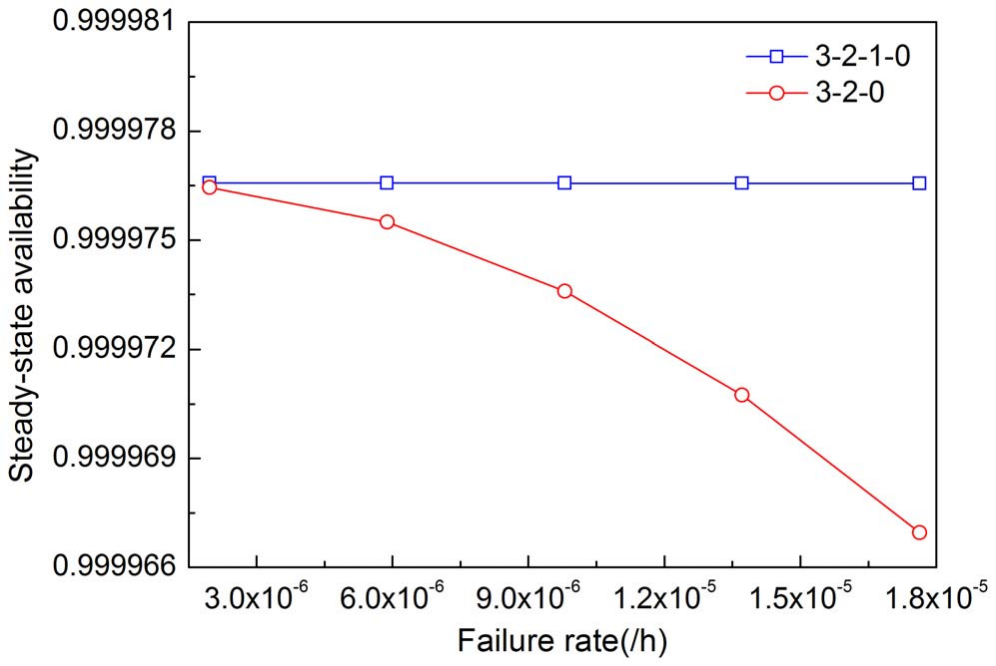
Effects of the failure rate of input module on steady-state availability in two schemes.

**Figure 6:**
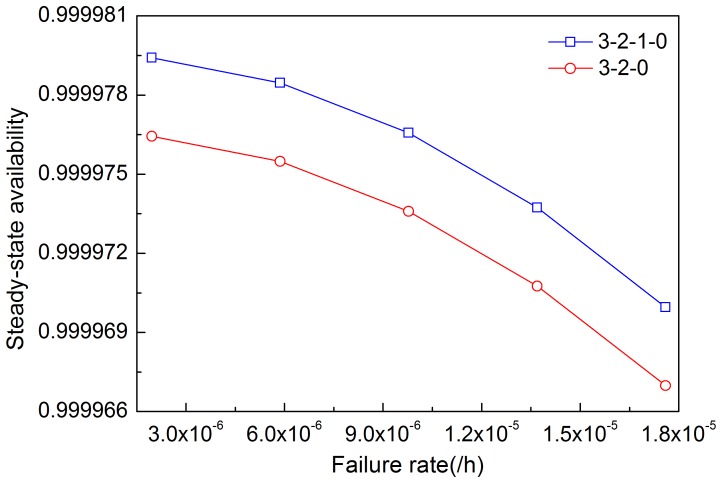
Effects of the failure rate of output module on steady-state availability in two schemes.

**Figure 7:**
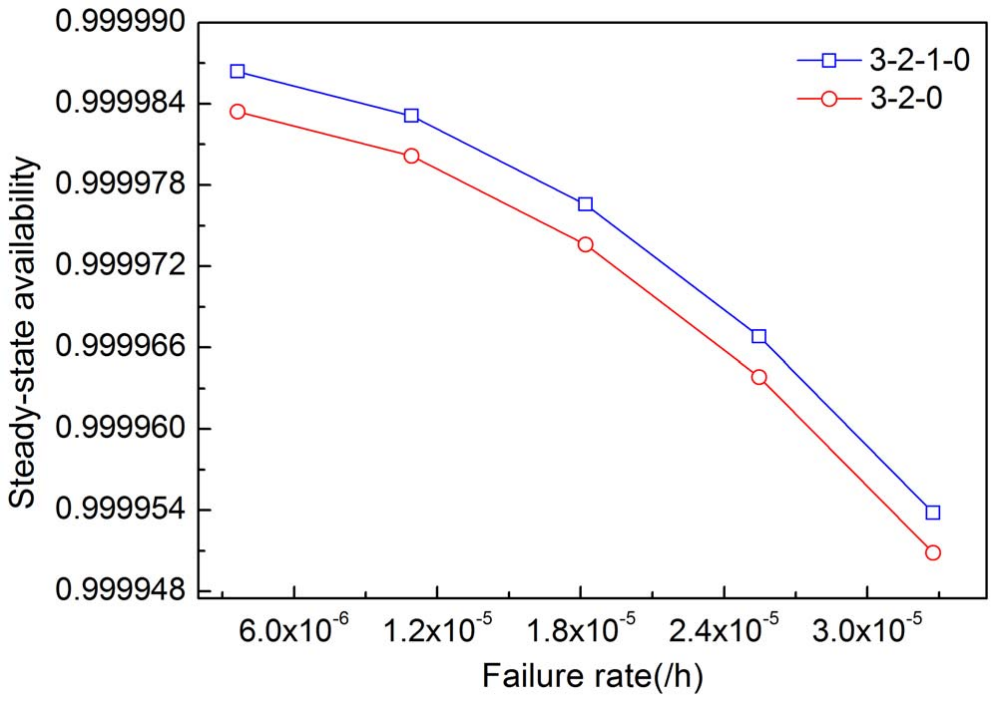
Effects of the failure rate of processor module on steady-state availability in two schemes.

**Figure 8:**
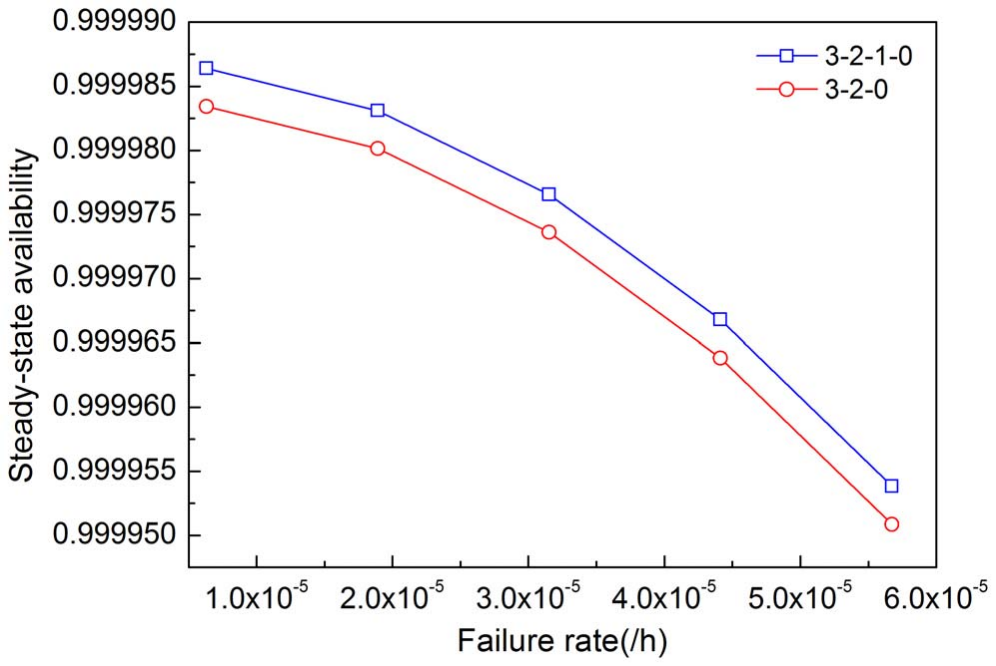
Effects of the failure rate of control panel on steady-state availability in two schemes.

Figures 5–8 illustrates the effects of modules' failure rates on the steady-state availability of the system in the two input voting schemes adopted in this work. With the same failure rate value, steady-state availability in the 3-2-0 input voting scheme is lower than that in the 3-2-1-0 voting scheme. As shown in Figure 5, steady-state availability decreases slowly in the 3-2-1-0 scheme, but rapidly in the 3-2-0 scheme, as input module failure rate increases. Hence, steady-state availability of the system is more sensitive to the input module in the 3-2-0 scheme compared with the 3-2-1-0 scheme. For other modules shown in Figures 6–8, the variation trends of steady-state availability in these two schemes are similarly connected with sensitivity. As the failure rates increase, the steady-state availability decreases.

**Figure 9:**
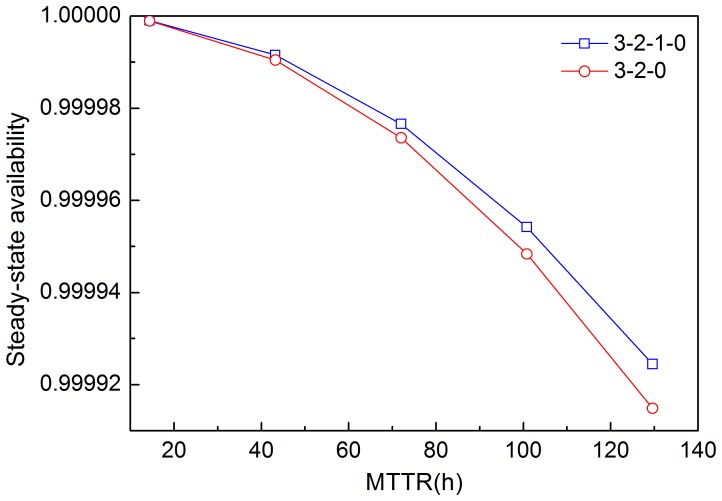
Effects of the MTTR on system steady-state availability in two schemes.

Mean time to repair (MTTR) is the average time required to repair a failed component or system. The reciprocal of repair rate *µ* is MTTR. As availability is related to repairs, the effects of MTTR change on system availability are plotted in Figure 9. MTTR varies by 20% to 180% of the original value in this paper. Figure 9 shows that the steady-state availability decreases as MTTR increases for both schemes. However, the 3-2-0 voting scheme is more sensitive to MTTR change, which indicates that the availability of the 3-2-0 scheme is more easily influenced by MTTR than that of the 3-2-1-0 scheme.

### Reliability

**Figure 10:**
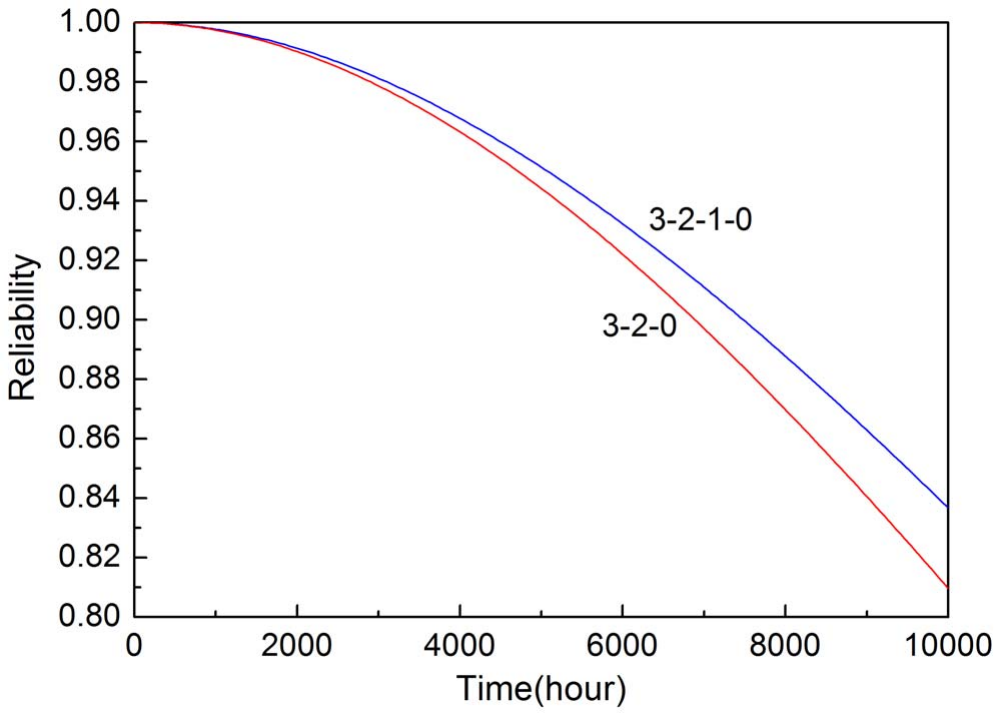
Reliability of the systems in two input voting schemes.

Based on Eqs. (3) and (7), reliability of the system with two input voting schemes is shown in Figure 10. It shows that reliability decreases over time and reliability of 3-2-0 input voting scheme decreases more quickly than that of 3-2-1-0 scheme. The 3-2-1-0 voting scheme has higher reliability than 3-2-0 voting scheme.

### MTTF

**Figure 11:**
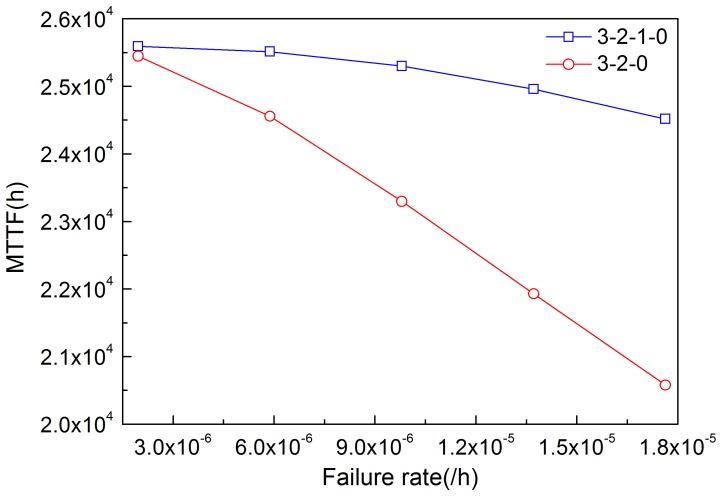
Effects of the failure rate of input module on MTTF in the two schemes.

**Figure 12:**
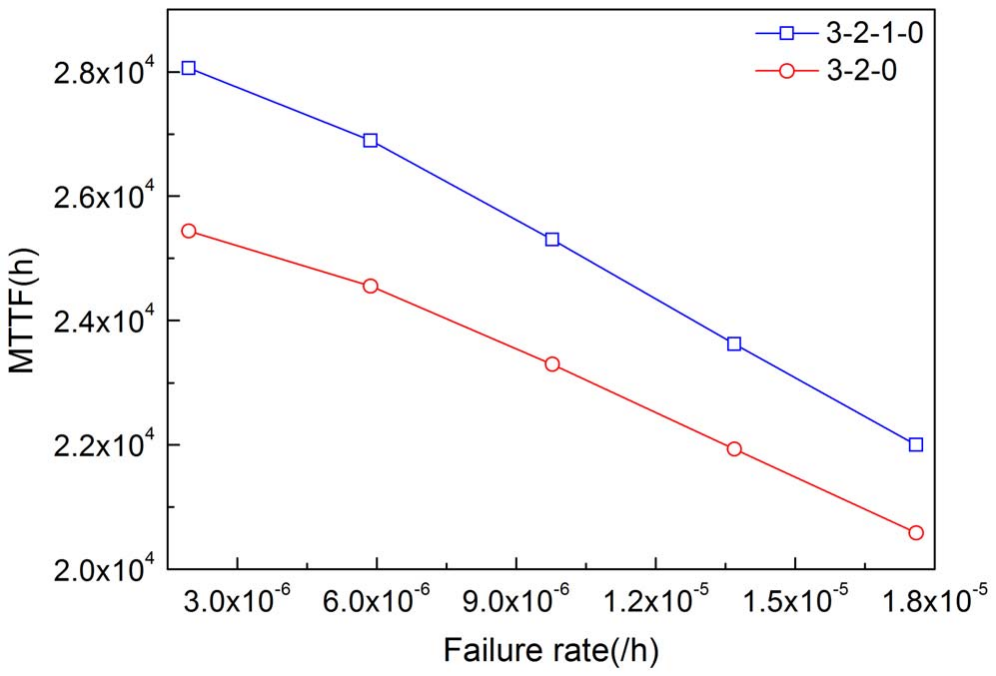
Effects of the failure rate of output module on MTTF in the two schemes.

**Figure 13:**
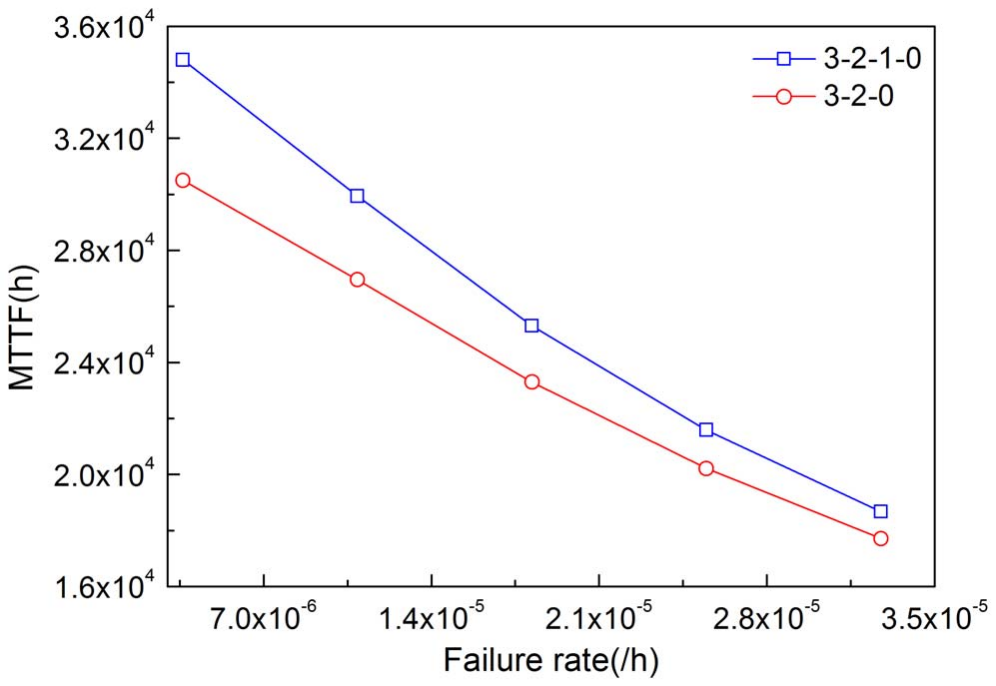
Effects of the failure rate of processor module on MTTF in the two schemes.

**Figure 14:**
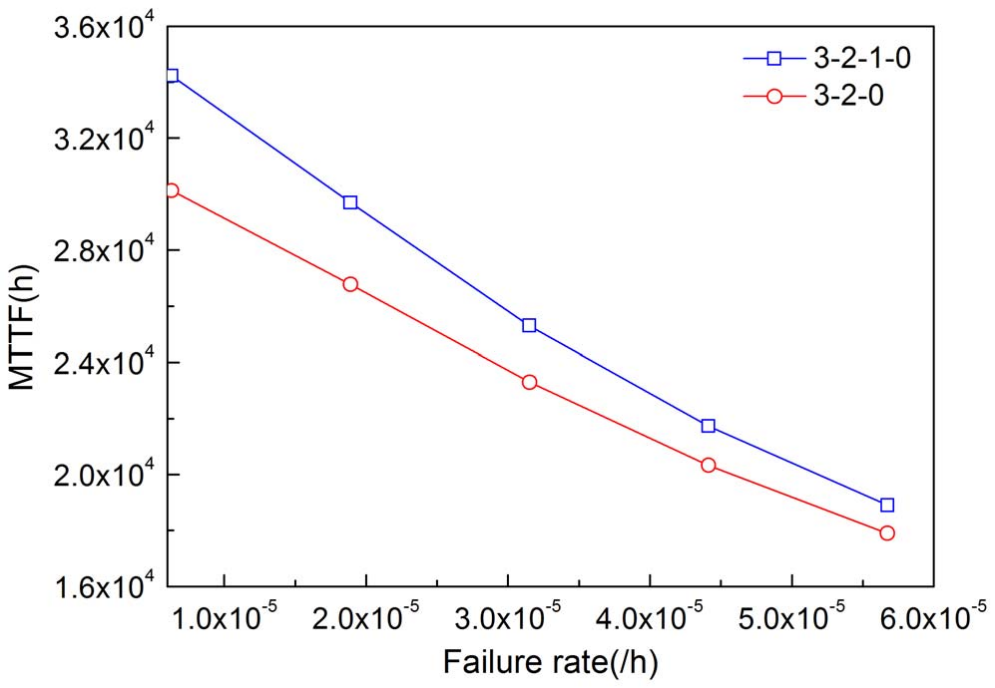
Effects of the failure rate of control panel on MTTF in the two schemes.

By solving Eqs. (8)–(10), MTTF can be obtained. Figures 11–14 illustrate the effects of modules' failure rates on MTTF of the system in the two input voting schemes adopted in this work. With the same failure rate value, MTTF in the 3-2-0 input voting scheme is lower than that in the 3-2-1-0 voting scheme. For the input module, as shown in Figure 11, MTTF decreases more rapidly in the 3-2-0 voting scheme as the failure rate rises. Input voting schemes greatly affects MTTF of the system with high failure rate of input module. For the other components, MTTF decreases in both schemes as the failure rates increase. Figure 13 and 14 show that the processor module and control panel module have similar influences on MTTF of the system in both schemes.

## Conclusions

This paper presents Markov models for the reliability analysis of subsea BOP electrical control systems. The effects of the 3-2-1-0 and 3-2-0 input voting schemes on reliability indices of the system are examined. In addition, the effects of failure rates of modules and repair time on system performance are also presented.

1. Compared with the 3-2-0 input voting scheme, the 3-2-1-0 input voting scheme has higher availability and reliability. From the aspect of high reliability, the 3-2-1-0 scheme is recommended for use.
2. For the both input voting schemes, the input module has the most different influence on system steady-state availability and MTTF. Processor module and control panel have similar influences on system performance with the two schemes. If the 3-2-1-0 voting scheme is applied, the input module will affect the system performance most slightly compared with the other modules.
3. The steady-state availability of the system decreases as MTTR increases. The 3-2-0 voting scheme is more sensitive to the change of MTTR than the 3-2-1-0 scheme. Therefore, if MTTR is reduced, the availability of the 3-2-0 scheme can obviously increase.
